# Monitoring quality and coverage of harm reduction services for people who use drugs: a consensus study

**DOI:** 10.1186/s12954-017-0141-6

**Published:** 2017-04-22

**Authors:** Lucas Wiessing, Marica Ferri, Vendula Běláčková, Patrizia Carrieri, Samuel R. Friedman, Cinta Folch, Kate Dolan, Brian Galvin, Peter Vickerman, Jeffrey V. Lazarus, Viktor Mravčík, Mirjam Kretzschmar, Vana Sypsa, Ana Sarasa-Renedo, Anneli Uusküla, Dimitrios Paraskevis, Luis Mendão, Diana Rossi, Nadine van Gelder, Luke Mitcheson, Letizia Paoli, Cristina Diaz Gomez, Maitena Milhet, Nicoleta Dascalu, Jonathan Knight, Gordon Hay, Eleni Kalamara, Roland Simon, EUBEST working group, Sabrina Molinaro, Michela Franchini, Valeria Siciliano, Elisa Benedetti, Marco Perduca, Alban Ylli, Gregorio Barrio Anta, Maria José Bravo Portela, Iciar Indave, József Rácz, Tomáš Zábranský, Michaela Štefunková, Percy Fernandez Dávila, Maris Salekesin, Sigrid Vorobjov, Monica Dan, Cristina Fierbinteanu, Dan Popescu, Ludmila Verdes, Adrian-Octavian Abagiu, Angelos Hatzakis, Maria Moudatsou, Tzanetos Antypas, Agnes Cadet-Tairou, Anne Marie Collins, David Liddell, Catherine Comiskey, Carla Rossi, Paul Griffiths

**Affiliations:** 1 European Monitoring Centre for Drugs and Drug Addiction (EMCDDA), Praça Europa 1, Cais do Sodré, 1249-289 Lisbon, Portugal; 2 Department of Addictology, First Faculty of Medicine, Charles University and General University Hospital, Prague, Czech Republic; 3 National Institute for Mental Health, Prague, Czech Republic; 4 Uniting Medically Supervised Injecting Centre, Sydney, Australia; 5 Marseille Univ, INSERM, IRD, SESSTIM, Marseille, France; 6 ORS PACA, Marseille, France; 7 Institute of Infectious Disease Research, National Development and Research Institutes, New York, USA; 8 Centre d’Estudis Epidemiològics sobre les Infeccions de Transmissió Sexual i Sida de Catalunya (CEEISCAT), Agència de Salut Pública de Catalunya (ASPC), Barcelona, Spain; 9 Consortium for Biomedical Research in Epidemiology and Public Health (CIBERESP), Madrid, Spain; 10 Program of International Research and Training, National Drug and Alcohol Research Centre, The University of New South Wales (UNSW), Sydney, Australia; 11 Health Research Board, Dublin, Ireland; 12 School of Social and Community Medicine, University of Bristol, Bristol, UK; 13 CHIP, Rigshospitalet, University of Copenhagen, Copenhagen, Denmark; 14 Barcelona Institute of Global Health (ISGlobal), Hospital Clinic, University of Barcelona, Barcelona, Spain; 15 National Monitoring Centre for Drugs and Addiction, Prague, Czech Republic; 16 Julius Center for Health Sciences and Primary Care, University Medical Center Utrecht, Utrecht, The Netherlands; 17 Centre for Infectious Disease Control, National Institute for Public Health and the Environment, Bilthoven, The Netherlands; 18 Department of Hygiene Epidemiology and Medical Statistics, Medical School, National and Kapodistrian University of Athens, Athens, Greece; 19 Spanish Field Epidemiology Training Program (PEAC), National Centre of Epidemiology, Carlos III Health Institute, Madrid, Spain; 20 Department of Family Medicine and Public Health, University of Tartu, Tartu, Estonia; 21 Group of Activists on Treatments (GAT), Lisbon, Portugal; 22 Intercambios Civil Association and University of Buenos Aires, Buenos Aires, Argentina; 23 Alcohol, Drug, and Tobacco Division, Health and Wellbeing Directorate, Public Health England, London, UK; 24 Leuven Institute of Criminology (LINC), Faculty of Law, University of Leuven, Leuven, Belgium; 25 Centre for Global Governance Studies (GSS), Leuven, Belgium; 26 French Monitoring Centre for Drugs and Drug Addiction (OFDT), Saint-Denis, France; 27 The Romanian Association Against AIDS (ARAS), Bucharest, Romania; 28 Department of Health, Wellington House, London, UK; 29 Public Health Institute, Faculty of Education, Health and Community, Liverpool John Moores University, Liverpool, UK; 30 Trinity College Dublin, The University of Dublin, Dublin, Ireland; 31 Centro Studi Statistici e Sociali CE3S, Rome, Italy

**Keywords:** Substance abuse, People who use drugs/PWUD, People who inject drugs/PWID, Injecting drug users/IDU, Best practice, Harm reduction, Knowledge exchange, Interventions, Indicators, Coverage, Epidemiology, HIV, HCV, Monitoring, Evidence-based, Drug services

## Abstract

**Background and aims:**

Despite advances in our knowledge of effective services for people who use drugs over the last decades globally, coverage remains poor in most countries, while quality is often unknown. This paper aims to discuss the historical development of successful epidemiological indicators and to present a framework for extending them with additional indicators of coverage and quality of harm reduction services, for monitoring and evaluation at international, national or subnational levels. The ultimate aim is to improve these services in order to reduce health and social problems among people who use drugs, such as human immunodeficiency virus (HIV) and hepatitis C virus (HCV) infection, crime and legal problems, overdose (death) and other morbidity and mortality.

**Methods and results:**

The framework was developed collaboratively using consensus methods involving nominal group meetings, review of existing quality standards, repeated email commenting rounds and qualitative analysis of opinions/experiences from a broad range of professionals/experts, including members of civil society and organisations representing people who use drugs. Twelve priority candidate indicators are proposed for opioid agonist therapy (OAT), needle and syringe programmes (NSP) and generic cross-cutting aspects of harm reduction (and potentially other drug) services. Under the specific OAT indicators, priority indicators included ‘coverage’, ‘waiting list time’, ‘dosage’ and ‘availability in prisons’. For the specific NSP indicators, the priority indicators included ‘coverage’, ‘number of needles/syringes distributed/collected’, ‘provision of other drug use paraphernalia’ and ‘availability in prisons’. Among the generic or cross-cutting indicators the priority indicators were ‘infectious diseases counselling and care’, ‘take away naloxone’, ‘information on safe use/sex’ and ‘condoms’. We discuss conditions for the successful development of the suggested indicators and constraints (e.g. funding, ideology). We propose conducting a pilot study to test the feasibility and applicability of the proposed indicators before their scaling up and routine implementation, to evaluate their effectiveness in comparing service coverage and quality across countries.

**Conclusions:**

The establishment of an improved set of validated and internationally agreed upon best practice indicators for monitoring harm reduction service will provide a structural basis for public health and epidemiological studies and support evidence and human rights-based health policies, services and interventions.

## Background

Important advances in interventions for people who use drugs (PWUD), in particular those who use opioids and people who inject drugs (PWID), have occurred over recent decades. Harm reduction services such as needle and syringe programmes (NSP) and opioid agonist therapy (OAT) [1] have been increasingly established, with 90 countries having NSP to some degree and 80 at least one OAT programme operational by 2016 [2]. This has contributed to reductions in viral infections (e.g. human immunodeficiency virus (HIV), hepatitis C virus (HCV)) and bacterial infections (e.g. tuberculosis (TB), sexually transmissible infections, skin infections), crime, overdose and mortality among PWUD. Health cost savings are being achieved globally, where harm reduction is in place, especially where these services are combined with antiretroviral therapy (ART), allowing millions of people living with HIV to stay healthy [3–15]. The provision of naloxone, a drug to reverse overdose, has expanded from paramedics to drug workers and to PWUD themselves and their peers [16–18]. Treatments for infectious diseases (e.g. HIV, hepatitis B virus (HBV)) and new direct-acting antiviral (DAA) treatments for HCV, when available, are having large effects on survival and quality of life and have opened new avenues for effective prevention [19–22]. Evidence on intervention best practice is mounting and is increasingly based on larger and better designed studies [23, 24].

Drug policies have also started to shift, even if the translation of evidence into policy remains difficult [25–28]. In some countries, there is cooperation between judicial and health authorities to mitigate harms associated with the criminalisation of drug use [29] and explicit or de facto decriminalisation of drug use [30–32]—these may often go together [33]—or even legalisation, in the case of cannabis [34–37]. Human rights-based approaches to drug treatment, incorporating harm reduction and social integration, have been implemented in a number of countries despite universal, national and global drug prohibition policies [38–40]. Despite such positive progress, however, many countries still have very low implementation levels of evidence-based programmes, exposing PWUD and the wider society to unnecessary health risks [13, 15, 41]. Above all, interventions appear to be frequently lacking for some of the most socially deprived groups, such as homeless, migrants, sex workers and prisoners [42–52]. Harm reduction and drug policy more widely have not been high on the international political agenda, with the United Nations General Assembly Special Session on the World Drug Problem in 2016 being the first high-level meeting after many years with the aim to debate drug policy. Also, the global target to reduce new HIV infections by 50% by 2015 was missed, and the latest UNAIDS (The Joint United Nations Programme on HIV/AIDS) report suggests that HIV infections among this group actually increased by one third between 2011 and 2015 [53].

Critically, there are still continuous gaps in information on how effectively interventions are actually being provided; their coverage, quality, client characteristics and the degree to which they fulfil the needs of different populations of drug users [13, 15, 54–56]. While in many countries there are regular—often costly—epidemiological studies on the characteristics and behaviours of drug users, the collection of comparable and reliable monitoring data on the extent and quality of routine interventions (for example NSP) and service implementation remains rare. Epidemiological studies and routine analysis of health indicator data are key to evaluating drug service effectiveness, but they are infrequently extended to and combined with detailed information on intervention characteristics [15, 41, 57–59]. A tight nexus between indicators of quality and drug service provision and health outcomes has been documented [60–62]. Despite the wide range of quality standards and best practice guidelines for drug services on the national and international level [6, 23, 24, 56], research has shown that adherence to these guidelines should not be taken for granted, and there is a need for data that reflect the reality of actual practice ‘on the ground’ [63, 64]. There is increasing interest in the quality and coverage of harm reduction services for people who use drugs and in the development of methodologies for measuring these [6, 56, 65]. An understanding of what services are being provided, in what form and the extent to which they are provided to individual users, including their views on the provision (where possible extending to enumeration of costs and if possible—in separate studies by specialist researchers—modelling of cost-effectiveness) is critical to the analysis of public health needs and whether these are adequately addressed.

This paper aims to identify which standardised data are needed—and why—for monitoring both the coverage and quality of harm reduction services [56]. This is not the type of research question that can be readily addressed through standard epidemiological methods. Rather, useful approaches may include analysis of historical developments in the area, critical discussion of current best practices (i.e. indicators in use that have proved successful) and data gap analysis.

## Methods

As a first step, we describe the historical development of established international monitoring systems and indicators in the field of drugs and health. We then propose a framework for further indicator development and evaluation in the area of harm reduction (and potentially other drug services, for examples see the footnotes below Table 3). This framework was developed using consensus methods, including nominal group meetings and email discussions [66, 67] reviewing existing quality standards [6, 56, 68], to capture and analyse the opinion and experience from a broad range of professionals/experts. The participating experts provided different perspectives and expertise (international and national monitoring system specialists, researchers, harm reduction professionals, government representatives) and included members of civil society organisations representing PWUD and people living with HIV/HCV. The framework lists candidate indicators for OAT, NSP and generic cross-cutting indicators for harm reduction (and potentially other drug) services. The framework with candidate indicators was developed in an iterative process of multiple commenting rounds until a stable consensus list of potential indicators (and areas for future indicator development) emerged. We discuss constraints (e.g. funding, ideology) and conditions for potential successful development of the suggested candidate indicators.

## Results

### Historical development of existing drug use monitoring systems

The global development of indicators in the drugs field was spearheaded in the area of HIV/AIDS. In 1989, one of the first common sets of indicators (behavioural) for people who inject drugs (PWID) was applied across countries by the World Health Organization (WHO) ‘13 cities study of drug injecting and HIV infection’ [69]. In 1998, the National Institute on Drug Abuse (NIDA), WHO and UNAIDS formed the ‘Global Research Network on HIV Prevention in Drug-Using Populations’ (GRN) to help control the HIV epidemic among PWID [70] by discussing best practice and exchanging national study methods and results in international meetings. The GRN was succeeded in 2004 by the ‘Reference Group to the United Nations on HIV and Injecting Drug Use’, a network funded by UNODC, WHO and UNAIDS, to estimate the global spread of HIV among PWID [71–73] and intervention coverage [13] using common methodology, which culminated in UN guidance for countries to set targets for intervention coverage [6, 74] and implementation [75]. Ongoing global monitoring has more recently been taken up by UN reporting systems [76, 77] and non-governmental and academic organisations [2, 78].

In Europe, comparable work on drug use started in 1982 with the ‘Multi-city study of drug misuse in Europe’ [79]. This expert network developed epidemiological indicators to interpret trends in drug use and their consequences from routine sources and studies across countries, leading to the first pan-European drug treatment data monitoring protocol [80, 81]. European multi-country impact studies on HIV/AIDS and PWID followed in 1989–1993 [82–87], leading to an increased interest in preventing HIV transmission in prisons [88–90]. The growing global attention paid to HIV/AIDS accelerated the urgency to improve responses for PWID, leading to the creation of a single agency for the European Union (EU) in the area of drugs. Since 1995, the European Monitoring Centre for Drugs and Drug Addiction (EMCDDA) and its national partners (the ‘Reitox Network’ (Réseau Européen d ´Information sur les Drogues et les Toxicomanies) of National Focal Points, as well as multiple topic-specific expert networks, have collaborated to gather evidence on the situation of drugs and their consequences to support national policymaking [41, 91–97]. A central area of this work concerns the development of the five ‘key epidemiological indicators’ of drug use and its consequences (general population surveys, population size estimates of PWUD at high risk of (or already experiencing) negative consequences and that include hidden populations, infectious diseases—HIV and viral hepatitis, overdose deaths, treatment demand) (Table 1) [98–100]. Despite the difficulties of collecting reliable data at a pan-European level, [101–103] these are being relatively well reported (almost all countries reporting on most indicators, Table 1), and they have been followed at the global level [73, 104–106].

**Table 1:** Epidemiological indicators for people who use drugs being used at European Union level

Domain	Indicators	Countries, out of 30, reporting in 2011–2015^b^	Data type	Additional information
Prevalence of drug use in the general population^a^	Prevalence of lifetime use, last year use, last month use	25	%	Representative (household) surveys with breakdowns by drug, age, gender, complemented by school surveys in 15-16 year old students (ESPAD) http://www.espad.org/
High-risk drug use/problem drug use^a,c^	Population size estimates of high-risk PWUD including hidden populations (all, opioids, stimulants, PWID)	25	Rate/1000	Confidence intervals, estimation methods
Treatment demand^a^	Clients entering treatment	30	Counts	Breakdowns by ever previously treated, treatment type, prison, main drug, sex, age at treatment, age at first use, referral source, living status, education, labour status, route of administration, frequency of use
Overdose deaths^a^	Number of deaths, average age	30	Counts	Breakdowns by gender, toxicology, ICD code
Infectious diseases^a^	Notifications and prevalence of HIV/AIDS, HBV, HCV among PWID	Prevalence: HIV 29 HCV 25, HBV 18–16; notifications: HIV/AIDS 30/29	Counts, %	Prevalence among young and new PWID
Seizures of drugs	Number, quantity in kg	28, 30	Counts, weights	Seizures by drug class, cannabis plants, tablets/doses
Price, purity/potency	Price, potency/purity	29, 29	Euro/g, % (%THC)^e^	Sample size, summary statistics, composition (% MDMA^d^/(meth)amphetamines)
Drug use in prison	Prevalence of lifetime use, last year use, last month use	10	%	Breakdowns by: before/in prison, drug class
Drug law offences	Number of: offences, offenders, either	25, 21, 30	Counts	Breakdowns by type (use, supply), drug class

^a^Five ‘key epidemiological indicators’. Available at http://www.emcdda.europa.eu/data/stats2016

^b^Year of reporting data to EMCDDA–the actual study year (year of primary data collection) is mostly 1 year earlier

^c^This key indicator has been renamed from ‘Problem Drug Use’ (definition: ‘injecting drug use or long duration/regular use of opiates, cocaine and/or amphetamines’) to ‘High Risk Drug Use’ (definition: ‘recurrent drug use that is causing actual harms (negative consequences) to the person (including dependence but also other health, psychological or social problems), or is placing the person at a high probability/risk of suffering such harms’). It attempts to define and estimate the population size of those PWUD that are likely to be in need of services due to having (a high risk of) negative consequences from their drug use, such as PWID or people who use opioids

^d^3,4-Methylenedioxymethamphetamine (‘ecstasy’)

^e^Tetrahydrocannabinol

A smaller number of intervention indicators were also developed, in the areas of drug treatment and harm reduction (Table 2). These concern both the provision of services (counts of clients entering treatment or syringes and clients/contacts in NSP) as well as coverage indicators (provision divided by estimates of the population in need of the service) [15, 107–109]. In 2013, a majority of countries were able to provide most of the provision indicators. However, reporting of the coverage indicators was significantly weaker, mainly because they necessitate additional information, in the form of population size estimates for PWUD as their denominators (from Table 1) (Table 2). Although provision indicators are important, for example to follow trends over time, they have inherent limitations, and additional coverage indicators are essential.

**Table 2:** Health and social intervention indicators for people who use opioids and people who inject drugs being used at European Union level

Intervention	Indicators	Countries, out of 30, reporting in 2011–2015^a^	Data type	Additional information
Provision				
Drug treatment (total)	All clients	30	Counts	–
OAT	All clients, by OAT medication	30, 30	Counts	Legal framework/providers
NSP	Syringes provided, clients, contacts, fixed sites, outreach sites	25, 19, 20, 28, 26	Counts	Estimated reporting coverage (%), NUTS2/3 level^b^
Coverage				
OAT	OAT clients divided by the estimated number of opioid users (Fig. 1)	20	%	Confidence intervals, estimation methods
NSP	Syringes provided divided by the estimated number of PWID (Fig. 2)	14	%	Confidence intervals, estimation methods

Available at http://www.emcdda.europa.eu/data/stats2016

^a^Year of reporting data to EMCDDA–the actual study year (year of primary data collection) is mostly 1 year earlier

^b^Nomenclature of Territorial Units for Statistics

Rates of drug use or drug injection differ strongly between countries, and thus, the comparability and interpretability of the simpler provision indicators (as counts, or rates per general population) may be seriously compromised with regard to the target populations of people who use opioids or PWID. Nevertheless, coverage indicators clearly also have limitations, for example uncertainty intervals around central estimates are often large and estimation methods not uniform, in addition to the lower reporting rates [41].

**Fig. 1:**
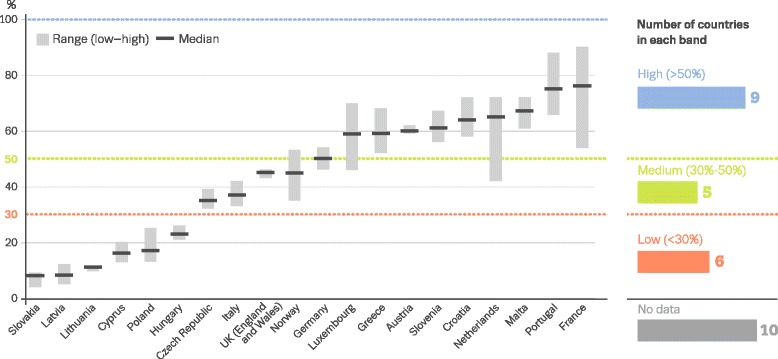
Estimated percentage of people who use opioids receiving opioid agonist therapy during 1 year (EMCDDA 2016) [41]. Note: data displayed as uncertainty intervals and point estimates. Estimates are based on latest data available on clients in opioid use treatment (2012–2014) combined with most recent estimates of opioid use prevalence (2007–2014). *Below red dotted line*, low (<30%); *between red and green dotted lines*, medium (30–50%); *above green dotted line*, high (>50%)

**Fig. 2:**
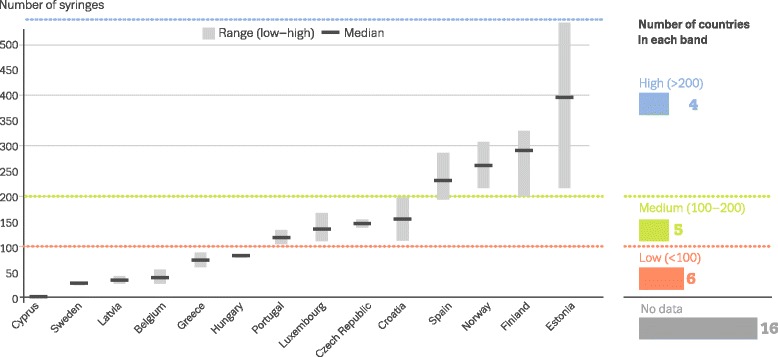
Estimated number of syringes provided annually through specialised programmes per person who injects drugs (EMCDDA 2016) [41]. Note: data displayed as uncertainty intervals and point estimates. Estimates are based on latest data available on syringe provision (2013–2014) combined with most recent estimates of PWID prevalence (2008–2014). *Below red dotted line*, low (<100); *between red and green dotted lines*, medium (100–200); *above green dotted line*, high (>200).

However, despite the significant drawbacks, they provide relatively comparable evidence (‘best available estimates’) across countries with regard to whether services meet the needs of the target population, with recent data suggesting that important differences in coverage may exist between countries in Europe (Figs. 1 and 2). These coverage indicators have been adopted at global level to assess policy implementation in the drug field [6, 13, 41, 74]. At the same time, it is clear that they are limited in terms of giving insight into modalities of provision and the perspectives of people using the service; thus, developing additional indicators of service quality is likely to improve the usefulness and interpretability of the intervention coverage indicators. Existing quality standards [6, 56, 68] provide an important basis for developing epidemiological indicators of service quality.

### Results of the expert group consultation

During 2014 and 2015, an international expert network began discussions to advance the monitoring and evaluation of best practice in drug-related interventions in Europe. It recommended focusing on the monitoring of coverage and quality of harm reduction services, as a first step to improving best practice implementation of wider drug services. This could best be achieved by integrating a limited set of additional indicators into the existing intervention indicators as currently coordinated by the EMCDDA as well as strengthening the reporting of existing indicators. Any additional indicators would then benefit from the ongoing efforts by European countries to ensure the timeliness, quality and completeness of data. Candidate indicators should compare key aspects of intervention delivery across countries, should be relatively easy to collect, where possible be evidence-based and, if not, based on expert consensus, and represent quality and coverage of services [110]. It was decided to start in a pragmatic way by producing a ‘framework’, i.e. mapping a list of potentially suitable candidate indicators and areas for future indicators, building on existing quality standards [6, 56, 68], the available expert opinions and experience and using consensus methods, as described above. The candidate indicators were chosen on their potential to reflect the structural and procedural quality of harm reduction services and service coverage [6, 56]. In future work, similar indicators could be set up for other interventions for PWUD, e.g. antiviral therapy or infectious disease testing [5, 6, 111]. For the suggested framework with candidate indicators of harm reduction service quality and coverage (OAT, NSP and ‘generic cross-cutting’ indicators), see Table 3.

**Table 3:** Framework for the development of indicators for quality monitoring of harm reduction services, with a focus on opioid agonist therapy (OAT) and needle and syringe programmes (NSP); priority indicators are in italics

Specific OAT indicators may include^a^:
*Coverage of estimated opioid user population (%, see Fig.* *1* *)*
*Waiting time to first treatment admission (months)*
*Methadone/buprenorphine dosage (grams)*
*OAT available (including new initiation) in prisons (in all /in some /no)*
OAT medicine covered by state /health insurance (yes /partly /no)
Illicit drug consumption tolerated (after dose induction phase) (yes /no)
Diagnosis or detailed assessment of current substance use, individualised treatment planning (yes /no)
Take home OAT available (yes /no)
Counselling required (yes /no)
Specific NSP indicators may include^a^:
*Coverage of estimated PWID population (syringes /PWID /year, see Fig.* *2* *)*
*Annual number of needles /syringes distributed and collected (administrative data, and /or estimated by weight)* ^*b*^
*Provision of drug use equipment and injecting paraphernalia (including for non-injected use e.g. foils for heroin chasing, stems and filters for crack smoking) (in all /in some /no)*
*NSP available in prisons (in all /in some /no)*
Coverage of all undertaken injections (syringes /100 injections)
Restrictions in numbers of syringes distributed per contact (yes /no)
Type of syringes (% low dead space, acceptance by users)
Modality (specialised NSP, outreach, pharmacy, other, e.g. drug treatment service)
Brief opportunistic motivational interventions provided (yes /no)
Generic cross-cutting indicators for harm reduction (and other drug services) may include^a^:
*Infectious diseases counselling, testing, vaccination and referrals (e.g. HIV, HCV, HBV, TB) (in all /in some /no)*
*Take away naloxone provided (in all /in some /no)*
*Information provided on safer use, injecting and safer sex (in all /in some /no)*
*Condoms provided (in all /in some /no)*
Accessibility: opening times and geographic coverage, outreach activities, costs to clients, no age limits, no parental consent requirements, targeted programmes for special populations (e.g. (pregnant) women, sex workers, underage users) (to construct overall index score: high /medium /low)
Integration /cooperation with other services and continuity of care: e.g. shared location /referrals to NSP, OAT, infectious diseases counselling and testing, antiviral and other medical treatment and care, overdose prevention, social support, housing, education, employment services (in all /in some /no)
Regular consultation with law enforcement /community /neighbourhood: avoiding nuisance and conflict, improving safety for both clients and community (index: high /medium /low)
Regular consultation with the users of the service: feedback, evaluation, client satisfaction (index: high /medium /low)
Assessment procedures: risk behaviours, needs, health status, informed consent, data confidentiality, written client records (index: high /medium /low)
Psycho-social interventions provided (with or without medication): (yes /no)
Frequency of contact with a counsellor /social worker (times per month)
Staff qualification, multidisciplinarity, education and (ongoing) training (index: high /medium /low)
Case /contact management follows protocol /guidelines (yes /no, specify which)
Type of funding source: private /public; national /international, etc.; and security of funding (per client, grant-based, etc.), utilisation monitoring (treatment slots used), peer support /aid (to construct an overall index score on funding continuity and reliability: high /medium /low)

^a^The quality indicators listed are mostly structural and procedural [56]. Outcome indicators are limited to OAT and NSP coverage estimates. Other outcome indicators may be considered (e.g. client retention and return rates, reductions in drug use, crime, improvements in health, etc.), but given their complexity, this may be more appropriate to assess in detailed service evaluation studies at national or local level [171] (although note [110]). Further work may be needed to link up more strongly with recently adopted EU quality standards [68]. Other harm reduction and drug interventions to be considered for monitoring may include antiviral and antibacterial therapy (e.g. HIV, HCV, HBV, TB), heroin-assisted treatment, drug consumption rooms/safer injecting facilities, testing drug content and handing out water at rave parties and similar events, police interactions with drug users affecting service utilisation, interventions in special settings (e.g. prisons, mobile or outreach interventions), social interventions, e.g. relating to children or family of PWUD, and monitoring and may even extend to drug policy indicators (e.g. minimum quantities of drugs allowed for personal use, sentencing practise, medical use of cannabis, decriminalisation/liberalisation of drug laws, drug treatment regulations, e.g. allowing opioid agonist therapy through primary caregivers), continuity of care following prison release or treatment discharge

^b^Measuring infection rates in returned syringes may form an important and cost-effective method for monitoring prevalence and incidence of infection in the population [135, 136].

Measures of central tendency (e.g. mean, median) may be complemented by measures of variability (e.g. range, interquartile range) to better capture intra- and inter-national variation.

### Framework of potential indicators and areas for consideration

As expected, two main interventions were indicated by the experts as central to harm reduction (mainly, prevention of infectious diseases such as HIV and viral hepatitis and of opiate-related overdose), namely, NSP and OAT. Other areas in harm reduction for further consideration of indicator development, but for practical reasons not included among the recommended indicators, included ART (both for HIV and viral hepatitis), consumption rooms and heroin-assisted treatment (Table 3). Under the specific OAT indicators, priority indicators included ‘coverage’, ‘waiting list time’, ‘dosage’ and ‘availability in prisons’. For the specific NSP indicators, the priority indicators included ‘coverage’, ‘number of needles/syringes distributed/collected’, ‘provision of other drug use paraphernalia’ and ‘availability in prisons’. Among the generic or cross-cutting indicators proposed for harm reduction services (and potentially other drug services), the priority indicators were ‘infectious diseases counselling and care’, ‘take home naloxone’, ‘information on safe use/sex’ and ‘condoms’ (for details, see Table 3).

## Discussion

This consensus study provides a basis for the development and implementation of indicators of harm reduction quality and coverage and highlights further areas of potential monitoring of best practice intervention. Twelve priority candidate indicators were identified, on OAT, NSP and generic service quality aspects. Most of these seem relatively easy to monitor, consisting of simple ‘yes/no’ responses or a basic statistic. We propose conducting a pilot study to test the feasibility and applicability of the proposed indicators before their scaling up, to evaluate their effectiveness in comparing service quality across countries. From the experience in Europe, we suggest that this development should be collaborative (‘bottom-up’) making use of national and local experience and involving a broad range of experts and stakeholders (e.g. professionals, policymakers, representatives of people who use drugs and/or drug services, harm reduction organisations) across countries [56].

Important services were not included for monitoring, e.g. ART, mainly due to difficulties in finding a simple operationalisation or a key statistic from routine data that is readily available for all countries to be reported (such data may be obtained by special surveys; however, these are costly). While NSP and OAT are services that are specific for people who use opioids or PWID, respectively, and thus client numbers can be interpreted more easily, for ART this is not the case and in practice it is harder to come by reliable numbers for specific at-risk groups in treatment, e.g. PWID or men who have sex with men. Other services that are important but were not included are heroin-assisted treatment, drug consumption rooms/safer injection facilities, drug testing and water provision at rave parties, police interactions with drug users and interventions in special settings such as prisons. Again, their non-inclusion resulted not because they were considered unimportant but rather they were thought to be harder to monitor (e.g. police interactions) or to be partly overlapping with other indicators (e.g. safer injection rooms with NSP). However, indicators not included here might still be considered for implementation by individual countries depending on national context and priorities. For example, in many Latin American and Caribbean countries, stimulant use is more important than opioids, which might require adapting the indicators [2, 112]. Our approach might be extended to areas surrounding the actual implementation of drug services. For example, drug policy indicators could be considered for monitoring, e.g. sentencing practices and minimum quantities of drugs allowed for personal use, decriminalisation/liberalisation of drug laws or drug treatment regulations may have profound impact on health and well-being of PWUD. A recent study proposed a framework to classify countries by their models of ‘governance of addictions’ from an analysis of national drug strategies [33]. Monitoring both drug policies and their actual implementation and practice might reveal important discrepancies between the two, providing key policy relevant information [113, 114].

Indicators for the quality of drug services must be closely linked to epidemiological data and methods. The development of OAT and NSP coverage indicators (Figs. 1 and 2) was made possible by the increased availability of routine epidemiological monitoring data and the increased use of statistical modelling methods. The methods to estimate population sizes of PWUD/PWID originated in biology and continue to be improved for epidemiological application even if they have not essentially changed [97, 102, 105, 115–129]. Mathematical and statistical modelling has more generally been useful to improve our understanding of intervention effectiveness and cost-effectiveness as well as to give insight in potential epidemic courses and processes, thus providing some basis to evaluate interventions [95–97, 130–134]. Different types of intervention have been studied using mathematical models, such as impact of needle exchange programmes [135, 136], impact of behavioural changes [137] and impact of treatment on transmission [138, 139]. Recent studies suggest that molecular analyses of infectious diseases may also provide added value to epidemiological surveillance as a basis for evaluating interventions [48, 140–143]. Moreover, comprehensive reviews of epidemiological data (and intervention effectiveness and implementation) have been carried out to estimate the burden of disease and quality of life, providing a means to compare health and societal impact of interventions across different diseases including through cost-effectiveness analyses [144–148]. Indicators should not be limited to national-level data only. Having subnational breakdowns—by city or region—would be critical to understand within-country variation in epidemiological trends and intervention impact [149–152].

Apart from using the proposed indicators individually, they might be used for system-level evaluation to monitor and guide service integration and referral at national level. For example, it is important to use these indicators together to assess the comprehensiveness of harm reduction programming, given the evidence that harm reduction interventions are most effective when used in combination [138, 153]. Another example of a combined approach may be provided by a ‘harm reduction cascade’ model, similar to the recently proposed HIV or HCV care cascades [19, 154, 155], where the ‘flow’ of people who use drugs would be modelled through a tailored set of services, ranging from catering the needs of incidental or recreational users to those who inject drugs or are heavily dependent, and/or may have a range of health and social problems. The HIV and HCV cascade model enables the identification of gaps in health system performance by estimating the percentage of infected who know their status, percentage of those in care, percentage of those on ART and percentage of those with undetectable viral load/sustained virologic response. Care cascade indicators relate to the timely provision of ART for HIV and best medical practices for HBV, HCV and other diseases (endocarditis, methicillin-resistant staphylococcus aureus (MRSA), anthrax, TB, etc.) and might similarly be developed for drug prevention, treatment and harm reduction measures. Another example focuses on the interface between judicial and public health interventions. This includes the analysis of police interactions with drug users in the context of their service utilisation, policy indicators (e.g. minimum quantities of drugs allowed for personal use, sentencing practice, medical use of cannabis, decriminalisation/liberalisation of drug laws [37]) and the continuity of care following prison release [156, 157].

The feasibility of monitoring drug service implementation will depend on resources in countries and may therefore be more limited in low and middle income countries. However, where a country lacks the resources to implement and further develop these indicators, the proposed framework may be useful to document the absence of data in specific areas, even if in a rudimentary form (e.g. a binary ‘yes/no’ checklist). Monitoring performance should be evaluated only after several years of data collection using performance indicators such as the number of countries providing data and assessments of the credibility of the methods and sources behind the available data. In practice it may take many years to arrive at a high reporting rate with good quality data, and maintaining a long-term perspective is necessary. With respect to clinical services performance, which is evaluated by health insurance systems and/or national health authorities [158], monitoring drug services may pose specific difficulties due to their multi-disciplinary nature and as they may depend on different government and private entities and multiple funding sources. Service provision may thus depend on the type of service providers (public, private, non-governmental organizations including peer-driven initiatives, general medical practitioners), funding sources (central government, local and regional governments, social health insurance, private and other sources) and funding mechanisms (grants, treatment case, daily costs, fee for service or payment by result) [159]. Other aspects of funding might also impact on service performance, quality and outcomes—such as the way providers are chosen and the ways services are paid for, e.g. block grant, capitation, payment for activity or payment for outcome [160], although the evidence of how the funding provisions influence outcomes is mixed [161–163]. Additionally, disaggregated spending records could indicate whether programmes invest in adequate numbers of well-trained staff and procure quality commodities that meet the needs of the people accessing the service—all related to the quality of service provision. While we recommend monitoring harm reduction funding, this did not make it into the 12 priority indicators, as our focus has been on the service coverage and quality per se. While investment in itself would not denote quality, whether a programme is funded by government or an international donor can have implications for its sustainability that are important to monitor. There are several countries in Europe, as well as globally, facing issues with harm reduction sustainability and funding. It would be timely to consider a separate pilot study on the use of indicators relating to harm reduction spending.

There are several limitations to this analysis. While we were able to identify a set of priority candidate indicators using a consensus approach, we cannot at this stage present empirical evidence on the potential problems or advantages associated with implementation of these indicators. However, with the established, mostly epidemiological, indicators (Tables 1 and 2), this was a process of trial and error where a number of countries start jointly piloting such data collection using an agreed protocol, exchange experiences in regular working group meetings and improve quality and comparability of data collection practice, adjusting the protocol if necessary. A prior step could be to carry out specific literature reviews on each of the indicators; however, this was beyond the scope of our study. Also, we were unable to grade the information and suggestions obtained from our expert group by levels of evidence quality [164], again this was beyond the scope of our study, and given the broad area we cover would have not been feasible. If in a future step specific reviews are carried out on each indicator it would be important to attempt grading the evidence for each of them, although such evidence is likely to be scarce and in need of being generated. Our consensus approach was not a formal Delphi study and could as such be criticised. However, we did include various consensus methods (expert meetings, repeated email commenting rounds) [66, 67]. We believe it is unlikely the results would have differed much depending on the exact consensus approach, given that all participants agreed with the final version of framework and indicators. We have also not been able to identify clear candidate indicators for monitoring patient values and preferences regarding harm reduction services, although further work might well be able to define such indicators, as has been already attempted in drug treatment research [165–170]. Finally, the services here discussed and for which we propose to develop indicators are ‘services’ in the form of programmes that are established by governments or private professional organisations and run for the benefit of ‘society’ or, at least putatively, in the benefit of clients or patients. In organisational terms, these are top-down services. What is not discussed in this article is the array of self-financed or funded users’ groups and their activities both in helping each other and also in providing useful and needed critique of the top-down services and policies. There is clearly a need for further work on this area with strong involvement of the target populations and their organisational representatives that services are serving.

## Conclusions

We propose a framework for the further development of indicators of coverage and quality of harm reduction services, as a first step to improving best practice implementation in the drug field. This is based on the successful development of established monitoring systems and indicators, and an international consensus exercise. This framework might be especially of use for professionals in charge of monitoring and/or funding service implementation and quality at higher (e.g. national, international) levels of aggregation, in addition to providing some guidance at the local and individual service levels. From the framework, 12 priority candidate indicators emerge that are conceptually simple, likely suitable to be collected on a routine basis, and should provide comparable key evidence on the quality and coverage of opioid agonist therapy, needle and syringe programmes and generic drug service aspects. We propose conducting a pilot study to test the feasibility and applicability of the proposed indicators before their scaling up and routine implementation, to evaluate their effectiveness in comparing service quality across countries. The implementation of a limited set of validated and internationally agreed indicators for monitoring harm reduction service best practice will provide a stronger basis for future public health and epidemiological studies, in order to advance evidence-based health policy.

## Abbreviations

ART: Antiretroviral therapy
DAA: Direct-acting antivirals
EMCDDA: European Monitoring Centre for Drugs and Drug Addiction
EU: European Union
GRN: Global Research Network
HBV: Hepatitis B virus
HCV: Hepatitis C virus
HIV: Human immunodeficiency virus
MDMA: 3,4-Methylenedioxymethamphetamine (ecstasy)
MRSA: Methicillin-resistant staphylococcus aureus
NIDA: National Institute on Drug Abuse
NSP: Needle and syringe programmes
NUTS: Nomenclature of Territorial Units for Statistics
OAT: Opioid agonist therapy
PWID: People who inject drugs
PWUD: People who use drugs
Reitox: Réseau Européen d́Information sur les Drogues et les Toxicomanies
TB: Tuberculosis
THC: Tetrahydrocannabinol
UNAIDS: The Joint United Nations Programme on HIV/AIDS
WHO: World Health Organization
